# Between empowerment and self-discipline: Governing patients' conduct through technological self-care

**DOI:** 10.1016/j.socscimed.2018.07.043

**Published:** 2018-09

**Authors:** Dimitra Petrakaki, Eva Hilberg, Justin Waring

**Affiliations:** a Department of Management, University of Sussex, BN1 9SL, United Kingdom; b University of Sheffield, United Kingdom; c University of Nottingham, United Kingdom

**Keywords:** UK, Governmentality, Technology, Empowerment, Self-discipline

## Abstract

Recent health policy renders patients increasingly responsible for managing their health via digital technology such as health apps and online patient platforms. This paper discusses underlying tensions between empowerment and self-discipline embodied in discourses of technological self-care. It presents findings from documentary analysis and interviews with key players in the English digital health context including policy makers, health designers and patient organisations. We show how discourses ascribe to patients an enterprising identity, which is inculcated with economic interests and engenders self-discipline. However, this reading does not capture all implications of technological self-care. A governmentality lens also shows that technological self-care opens up the potential for a de-centring of medical knowledge and its subsequent communalization. The paper contributes to Foucauldian healthcare scholarship by showing how technology could engender agential actions that operate at the margins of an enterprising discourse.

## Highlights

• Digital health policy in UK assumes an enterprising & empowered patient identity.
• Tensions between empowerment & discipline occur as patients self-care digitally.
• Digital health creates space for agential actions that improve communal health.
• A health-making agency operates in the margins of governmental self-care discourse.

## 1 Introduction

Across the developed world, health policies encourage patients to take greater responsibility for their healthcare (Armstrong, 2014). Technological self-care refers, in this paper, to the ways patients are encouraged to use digital interfaces (e.g. health apps, online platforms) to manage their healthcare, including monitoring long-term conditions (e.g. diabetes), managing treatments (e.g. cancer), or making better healthcare choices (e.g. tooth brushing). Such technologies were central to English Department of Health's (DH) digitalization strategy for the National Health Service (NHS), which at the time of the research were in the process of validation. As we show, the potential of such technology goes beyond individual patient health to affect health research, clinical decision-making and service planning.

Digital health technologies increasingly feature in the reconstitution of patients as rational and reflexive agents (Adams, 2011), competently engaging in the digital management of their health (Lupton, 2014). Others interpret the use of digital technologies as re-constituting patient identities as the ‘producer’ or the ‘entrepreneur’ of health (Barrett et al., 2016; Crawshaw, 2012; Lupton, 2016b). This shift has generated debate on the interplay between empowerment and self-discipline (Lupton, 2016a; Schüll, 2016). Drawing on the work of Michel Foucault (1991a; 1991b; 2008), we argue there is an underlying tension between the empowerment of patients to care for themselves through digital technology, and the enrolment of patients in a self-disciplining ‘enterprising’ identity. This tension can be further explained as an attempt to enable individuals to better look after their health whilst also encouraging self-control. Individuals perform an enterprising identity as they become more responsible and accountable for what they are supposed to be doing (for example to self-care in order to reduce unnecessary health expenses) without being directly managed by others such as doctors. (Dean, 1999; Foucault, 2008). The growing significance of technological self-care, speaks to Foucault's concept of governmentality, especially for understanding how digital technologies operate as part of a wider apparatus of government and for engendering new self-governing patient identities (Lupton, 2016b).

Extant literature on governmentality in healthcare has mostly emphasised the disciplinary effects of health technologies (Crawshaw, 2012; Martin et al., 2013; McNay, 2009; Randall and Munro, 2010; Skinner, 2013; Waring, 2007; Waring et al., 2016), but overlooked the agential potential health technologies may also engender. Our study aims to offer a re-appraisal of Foucault's work specifically in relation to the possibilities for agency it opens up (Martin and Waring, 2018; McGivern et al., 2017). Drawing upon research into the digitalisation strategy of the English NHS, it analyses tensions between empowerment and self-discipline embodied in contemporary discourse of health policy makers, digital health technology experts and patient organisations around the ways in which technology can be used to enable individuals manage their conditions and self-care. The study shows that digital health technology ascribes an enterprising identity to patients that serves the economic needs of an enterprise health system, but also offers patient opportunities to participate in the production of new knowledge (in the form of online peer advice, co-design of devices, sharing of experience) that has effects for the broader health communities. We name this a health-making agency and show that it emerges from a de-centering and a subsequent communalisation of health knowledge. Although immanent to governmental discourse, this health-making agency is not necessarily or wholly subjected to it, but rather operates at the margins of an enterprising identity.

## 2 Empowerment & (self-)discipline in digital health governing

In the past few years a number of digital health interfaces, such as health apps, platforms and wearable devices emerged intended to enable better management of one's health (Barrett et al., 2016; Lupton, 2016a; Schüll, 2016; Tempini, 2015). These technologies expand the ‘medical gaze’ beyond the confines of the hospital into everyday life producing both empowering and disciplinary effects.

New technologies such as health apps enable the constant generation and transmission of data, which can be used to monitor health and well-being (Barrett et al., 2016; Kallinikos and Tempini, 2014; Lupton, 2016b). Data about, for example, heart rate, calorie intake, steps taken, or miles walked, are presented in more or less sophisticated ways to engender empowerment and behaviour change through constant self-surveillance (Lupton, 2014; Till, 2014; Ruckenstein, 2014). Patients' active participation in the collection of health data also enhances their understanding of their body and condition. In particular, patients can become more knowledgeable and have more control over chronic diseases that were previously seen as unmanageable or reliant on medical expertise (Lupton, 2016b). Furthermore, digital technology empowers patients by giving them the opportunity to organize online large communities around a health condition or service (Radin, 2006), and to use the gathered data to challenge health providers (Barrett et al., 2016; Griffiths et al., 2012; Radin, 2006; Tempini, 2015). These developments have the potential to empower the most medicalised patients (Klawiter, 2008), or to reverse the roles between experts and lay groups (Novas and Rose, 2000, p.490).

Various scholars caution against overstating patient empowerment by drawing attention to the new responsibilities imposed on the individual, through new technologies, ‘*to optimize ‘healthy’ bodies and minds*' (Wehling, 2011, p.227). Studies also show that patients can struggle to take on these responsibilities, often because of their physical or mental impairments (Hasselbladh and Bejerot, 2007), leading to an exclusion of those individuals who may be deemed to be more vulnerable or unable to exercise self-care (Ravn et al., 2016). Further, health technologies reduce health into abstract parameters and statistical representations that do not necessarily consider the sociological or biological factors of patients' lives. Digital health technology also enables the production of ‘big data’ (Kallinikos and Tempini, 2014; Tempini, 2015), which can lead to the formulation of new rules for ‘healthy’ conduct and, as Barrett et al. (2016) have shown, the creation of a knowledge-base of disease profiles that expand the potential of medical intervention and governing.

This paper suggests that the implications of digital health technologies for patients' self-care are better understood as located at the intersection of (self-)disciplinary regimes and the enterprising interpellation of governmental norms. Foucault's work on governmentality, and the work of his followers, shows the dynamic interplay between discipline and empowerment encapsulated in contemporary health discourse in neo-liberal digital governing (Foucault, 1991a; Randall and Munro, 2010; Vallas and Hill, 2012; Waring, 2007; Waring et al., 2016).

Foucault's work explores how social knowledge, as articulated through various discourses and technologies, defines the moral parameters for social conduct, and in so doing, constitutes the subjects of which it speaks (Foucault, 1988). His early works show how knowledge acts as a form of disciplinary power through its ability to define, classify and survey particular subjects (e.g. the mad, the criminal etc.) (Foucault, 2002).

His later works developed a more nuanced understanding of the relationship between power/knowledge that centred on the ‘conduct of conduct’ or the ways subjects are constituted to be active in the government of their own moral behaviours (Foucault, 2008; Rose and Miller, 1992). Foucault saw governmentality as embodying an economic rationality that extends the principles of the market to new fields of governing as a means of ‘veridiction’ that decides what is ‘right’ and ‘wrong’ in the degree and type of governmental intervention (Dean, 1999; Foucault, 2008). Under this new governmental lens, every individual operates as a phenomenally free self-contained enterprise in its exchanges with other individuals and institutions (Foucault, 2008). An ‘enterprise society’ is a society of competition and production (Foucault, 2008) which values individualism, flexibility, reflexivity and accountability (Vallas and Hill, 2012). Foucault (2008) explains that enterprising conduct is produced via a multitude of institutions that move responsibility for conduct from the state to the subject, and ensures the attainment of governmental goals without direct intervention (Adams and de Bont, 2007; Rose and Miller, 1992). Drawing on other elements of Foucault's writing (2008) there is growing interest in the contribution of pastoral power in the conduct of conduct, especially the way moral leaders contribute to subjectification through both guiding and overseeing individual behaviour (Martin and Waring, 2018). This suggests that empowerment is not an antithesis but a necessary condition of (self-)discipline (Dean, 1999; McNay, 2009; Vallas and Hill, 2012).

In the digital age, the enterprise society is encapsulated in the logic of the consumer as co-producer; an idea that has already been transferred to healthcare with patients taking more responsibilities to manage their condition (Crawshaw, 2012). Self-caring is central to an enterprising identity whereby patients are involved in the active interpellation (and not imposition) of normative regimes. Digital health technology can promote active ‘patienthood’ performed through continuous self-monitoring with the aim to take control over one's health and one's selfhood (Lupton, 2016a, 2016b). Patients' responsibilisation allows ‘active patients’ (Rose, 2007, p.11) to be proactively engaged in the promotion of their own health. This form of self-governing thus has strong normative connotations, which are not captured by an emphasis on disciplinary interventions alone.

Our study focuses on the ways patienthood can go beyond the performative aspects of an enterprising digital health discourse (Introna, 2016). More specifically, it aims to explore the tensions between empowerment and self-discipline that an enterprising digital health governing entails, with a focus on how health technology provides opportunities for acting at the margins of governmental discourse (McNay, 2009; Randall and Munro, 2010; Skinner, 2013).

## 3 Health technologies in an ‘enterprise’ health system

Our study focuses on the use of digital health technology in the English NHS. Since 2011 the DH reoriented its digital technology strategy from being a provider of technology to being a facilitator of a digital health market. In this role the DH sets minimum requirements on which technology is endorsed for use by the NHS (National Information Board, 2015). The approval procedure for new technologies relies upon developers' self-assessment, rather than central evaluation of content, suggesting the power of the market to determine what technologies are ‘right’ for self-care (Dean, 1999; Foucault, 2008).

The DH also aimed to instil a culture shift in the NHS based on the promotion of patients' ability to choose ‘*NHS-accredited health and care apps and digital information services*’ (DH & NIB, 2014, p.6). Policy makers argue that information technology is vital for patient choice (DH, 2012, p.11) and that it empowers people ‘*to take charge of their own health, by providing information, support and control*’ (DH & NIB, 2014, p.9). Self-management requires that patients develop expertise of their condition, and the skills to use technology to better manage their health (DH & NIB, 2014). Patients with chronic conditions are seen as becoming ‘*experts by experience*’ (NHS England, 2014a, p.12). Self-management of one's health is not presented as merely a way of administering a long term illness (e.g. monitoring blood pressure) but includes making informed choices, avoiding complications and staying healthy (NHS England, 2014a, p.12). Patients are seen as increasingly empowered ‘*co-producers*’ of their healthcare (National Information Board, 2014, p.4; also NHS England, 2014b, p.9); acting as enterprising participants that take on full responsibility for their health (Dean, 1999; Foucault, 2008; McNay, 2009).

The enterprising modality of patienthood relies on the economic efficiency of health information technology. Reports suggest that technology needs to be ‘*harnessed*’ and ‘*exploited*’ (DH & NIB, 2014, p.8), with health-related data amenable to ‘*extraction, collection, storage and transmission*’ (DH & NIB, 2014, p.15) with the goal of ‘*doing more for less*’ (DH & NIB, 2014, p.9). Significantly, empowering patients depends on patients' active assumption of self-responsibility, a sentiment advocated by the current Secretary of State for Health:

‘the best person to manage a long-term condition is the person who has that long-term condition. The best person to prevent a long term condition developing is not the doctor - it's you’ (Hunt, 2015).

In the context of the above discourse, our study explores the tensions between self-discipline and empowerment inscribed in these technologies and identifies the agential potential of technological self-care that goes beyond the confines of a health enterprising identity.

## 4 Research methodology

The paper draws upon an interpretive study grounded in a Foucauldian theory of patienthood (Alvesson and Deetz, 2000; Crotty, 1998). The study investigated how digital technologies, such as health apps and online platforms, are involved in patients' management of their health. A variety of technologies are intended for self-management of health, such as insulin pumps for diabetics or wearable sensors for patients suffering from dementia; this study focuses exclusively on digital interfaces designed primarily for patients' rather than clinicians' use. It focuses on developers who were funded by the DH to develop digital solutions that support self-care, broadly conceived. The technologies varied in terms of their expected frequency of use, purpose and health condition, including digital solutions for the everyday health management (e.g. tooth brushing); for the monitoring of a condition during treatment (e.g. breast cancer); and also for the management of chronic diseases (e.g. diabetes). Although these technologies are not comparable and do not generate the same type of data, they nevertheless emerge from the same enterprising health context and thus encapsulate an economic logic and a responsibilisation discourse, whilst revealing the potential range of uses and agency they can provide to patients.

The study draws from data collected between August 2014 and May 2016 and aims to explore 1) how key stakeholders, such as health policy makers, health technology experts and patient organisations, respond to the growing calls for patients to self-manage health by means of technology; and 2) the expected implications of technological self-care. Data was collected through documentary analysis and semi-structured interviews. We designed and conducted our research according to the research governance frameworks set by our institution. We received informed consent from all participants.

We collected and analysed 59 documents coming from a range of different sources including: health policy makers (31) such as DH, NHS England, Health & Social Care Information Centre (HSCIC), National Information Board; blogs and newspaper articles (15) such as National Health Executive, Cost of Living etc. and digital health technology experts (13), including developer documentation, patient surveys, evaluations etc. We selected documents on the basis of their relevance to the topic and the questions. We focused on documents that presented the digital health discourse, the strategy that designers, patient organisations and NHS put in place to implement policy, the design and functionality of apps and platforms and the use (actual or projected) of digital technologies. We excluded documents that contained technical specifications or detailed the development process of apps/platforms. We treated documents as texts inscribed with certain discourses and aimed to unpack and discuss them vis-à-vis existing knowledge coming from the review of the literature and interview transcripts (Alvesson and Skoldberg, 2000).

We conducted 31 interviews with three main stakeholder groups: health policy makers (8 interviews); representatives of patient organisations and patient-users of digital technologies (10 interviews); and digital health technology experts working on the design of apps and patient platforms (13 interviews). We initially identified key stakeholders on the basis of their involvement in the digitalisation strategy and market, whilst also consulting health policy makers and academic experts in this subject. For example, we interviewed health policy makers who were in charge of implementing and promoting the digitalisation agenda for the self-management of health (such as NHS England, National Institute of Clinical Excellence (NICE), National Data Guardian and HSCIC etc.). We also interviewed digital health experts who had been funded by the NHS to upscale, advance and improve their digital health technologies in support of this strategy. These technologies would be validated and endorsed by the NHS for subsequent use by healthcare providers and patients. The project also involved patient organisations that promoted self-care, such as Parkinson's UK, as well as other organisations supporting patients in the use of technology such as HealthWatch. Participants were also invited through recommendations from previous interviewees. Table 1 presents in more detail the organisations that participated in our study.

**Table 1:** Overview of organisations participating in the study.

Health policy makers	Patient Organisations	Digital health experts
NHS England	Patient Information Forum	Integrated change
NICE	PatientView	PxHealthcare
HSCIC	HealthWatch	MandTech
National Data Guardian	Meeting of Minds	DrDoctor
Digital Health and Care Alliance (DHACA)	Mylife	PatientJourney
	Parkinsons UK	BrushDJ
	Patients Know Best	Umotif
	Care Opinion	Mhabitat
		Cupris Health
		OutcomesBasedMedicine
		AliveCor
		Just Checking
		Painsense ADI

All interviews were conducted in person, with the exception of one telephone interview. We used different thematic guides to ensure questions were relevant to each stakeholder group. Interviews with policy makers focused on: the range of digital technologies intended for self-care; design requirements; expected benefits for NHS, care and patients; collaboration with technology designers; consultation with patient groups; views about the potential of patient-reported data; and patients' responsibility and choice. Interviews with digital health experts focused on how health apps and platforms work; benefits to the users; collaboration with DH or NHS England; assumptions made about patients as users of digital health technology; types and usage of data; and feedback from patients. Interviews with patient associations focused on the use of relevant digital health technologies; reasons for non-use, expected benefits; views on the NHS's digital health strategy, risks and challenges expected from it.

We analysed findings from interviews and documents following an iterative thematic process. We used NVivo to organize the coding process and to establish links between the different codes. Themes emerged when codes and their relations were refined and analysed through the literature outlined above with the one shaping the other (Alvesson and Skoldberg, 2000). Some of these themes included: patient empowerment; patients' interaction with digital health interfaces; health knowledge production; health apps design and use; patient feedback to providers etc. The analysis was inductive but from the outset was framed by a Foucauldian understanding of governing and subjectivity. Analysis allowed the emergence of unanticipated themes, such as the ‘health making agency’ and enabled some degree of saturation with consistent and repeated themes emerging across different stakeholder groups. Opposing views specifically between stakeholder groups enabled us to build a critical dialogue between the different views presented.

The next section presents our findings clustered around the tensions between the empowering and disciplinary effects embedded in the enterprising health discourse and the potential for agential action that may emerge at the margins of this entrepreneurial activity.

## 5 Findings

### 5.1 Empowering and disciplinary effects of an ‘enterprising’ health service

This section analyses how policy-makers and technology experts envisage digital health technologies as empowering patients to take greater responsibility for their health (Crawshaw, 2012; Rose, 2007; Lupton, 2014). For policy makers this ensures the inculcation of an economic rationality into (phenomenally) empowered entrepreneurial patients. We show however that in the operationalization of these discourses, as manifested by the design and expected use of digital health technology, parallel discourses emerge around wider societal imperatives such as population health, clinical research, and service planning. These are not necessarily conflicting discourses but are co-constitutive, suggesting that multiple rationalities can be encoded within technologies, offering space for multiple frames of action and possibilities for agency.

For policy-makers, digital health technologies promise a revolution in public health. They help to realise longstanding ambitions for more individualized healthcare where patients are empowered to take greater responsibility for their health and, by implication, become less dependent on government.

‘we all have to take a bit more responsibility and we all have to challenge ourselves in terms of our health behaviours and …adopt behaviours that are supportive of good health’ (Health policy maker).

Policy-makers’ expectations for health technologies have a dual concern, of empowering individuals and reducing professional responsibilities. This reflects an underlying economic agenda of restricting the economic burden of caring for the sick. Given the financial constrains on the NHS, self-care will in the future be an imperative.

‘…improve the lives of 3 million people through the use of technology-enabled care services (telehealth and telecare) by 2017, supporting people with long term conditions to manage and monitor their condition at home, and reducing the need for avoidable visits to their GP practice and hospital’ (NHS England, 2014b, p.32, p.32)

‘You will find a number of patients who don't like the idea of technology… The point is they've got two choices. The two choices are, do you now start working out how you are going to work with your doctor without seeing them so often or do you wait until the health care system collapses and you don't see your doctor at all… Unless you get your head round it now, there won't be anyone to look after you in future’ (DHACA).

This economic rationality permeates the reasoning of digital health experts. For example, the designer of an app to influence tooth-brushing behaviour focused on the potential cost savings to the NHS.

‘if you get someone just to spit the toothpaste and not rinse you reduce their risk of decay by 40%. If you do that over a population you can significantly reduce the disease … the cost of that disease in the UK you are looking at well, 3.4 billion pounds …. We know that 1.7 billion of that is on something that's preventable. It you get 1% in a billion pounds, it's a lot of money.’ (Health app designer).

For other digital health experts, discipline to technological self-care could in the future constitute a condition for accessing health services - inability to self-monitor could become a basis for exclusion. This adds to studies showing the marginalisation of patients who are unable or reluctant to perform expected roles (Crawshaw, 2012; Hasselbladh and Bejerot, 2007; Ravn et al., 2016).

‘It will enable the clinician to maybe the day before the appointment … check your record and [say] it doesn't look like you've done anything. There is no point coming in for a consultation with the NHS … if you are not prepared to self-manage yourself at home…’ (Health app designer).

An economic logic also guides the thinking of patient organisations that accept that the economic or commercial viability of the digital health market seems to be prioritized over the products' suitability to assist patients in taking care of themselves.

‘everybody wants to have something totally directly for them, but … this is supposed to be a business as well … they have to do something for a much broader group to be able to have any kind of return on your investments’ (Patient organisation representative).

Despite the popularity of this economic discourse, other digital health experts invoke a clinical discourse to emphasise the health benefits, not just to individual users, but to wider society. They refer, in particular, to the potential of digital health technology to generate large volumes of personalised health data that can be routinely scrutinized using data analytics to generate new insights into public health, the effectiveness of treatment, side effects, and patient adherence. Unlike data generated through expensive clinical trials and clinical expertise, aggregated health app data is described as offering real-time patient-reported data to inform service planning and public health interventions. As such, it affords not only discipline over individual patients, but a form of population-wide biopower for ‘*the maintenance of life and the wellbeing of the population*’ (Dean, 1999; p142).

‘…if we really wanted to make a difference in how patients are being treated, we needed to collect longitudinal data, but also the patient reported outcome data. For that purpose, we decided to build … a platform or mobile tools that can collect this type of data that we need for medical research to understand how patients really respond to treatments. … we collect and purely anonymise aggregated data for medical research to improve the treatment of cancer.’ (Health app designer).

Digital health experts saw the potential societal benefits as further motivating individual participation. The quote below demonstrates how designers frame their apps as contributing to the ‘greater good’ where patients' responsibilisation is inextricably linked to their power to generate meaningful data with potential social value. Implicit to this is, as we show below, the potential of technologies to afford novel forms of agency as individuals interact and use it.

‘We wanted to boost our recordings so we sent a note out to our users saying, did you know that by using this device daily you help us learn about heart health. We saw a tremendous boost in our recordings. People felt they were contributing. It wasn't just a meaningless trace’ (Health app designer)

An economic rationality governs health policy discourse on patient empowerment and this also affords alternate (clinical and societal) discourses to emerge oriented around the value of patient-produced data and a subsequent de-centring of medical expertise. Next section describes the operationalization of governmentality as individuals use technology to self-care and in doing so get involved in the production of health data.

### 5.2 Self-discipline through self-care & the empowering effects of health data

Health technologies provide patients with greater choice and empowerment over how to monitor their general well-being and proactively survey lifestyle behaviours and engage in personal health improvement. Prerequisite for this is that patients feed constantly health data into the technology, which are then re-presented as reports or graphs, giving back to patients recommendations for behaviour change. It is notable here how health advice is produced algorithmically, without the mediation of a medical expert. In this way, technology incites normalized ‘healthy’ behaviours and effects self-surveillance (Lupton, 2014; Till, 2014; Ruckenstein, 2014; Schüll, 2016).

‘We have some automated ways of telling them whether or not they should have to call a doctor or actually feel okay about their wellbeing’ (health app designer).

‘the feedback that you receive is ‘no pain’ and that's great..... ‘A lot of pain’ and please contact our pain management centre … But it's up to me to call them’ (Health app designer).

This element of patient choice over the advice they get from a health technology is crucial in the development of ‘structured freedom of action’. Health apps rarely interpret data, rather they collect and represent it back to patients. Interpretation would require the upgrade of a health app to the status of a medical device and would assume legal liability for the information it provides. Being unable to provide formal medical advice, health apps render patients even more responsible for interpreting and acting upon their data. A health app designer said, doctors cannot be in charge of the reports produced by an app, even if they have prescribed it, suggesting again the withdrawal of medical expertise from self-care. The grey area of responsibility is filled in by patients themselves, expanding the degree of ‘responsibilisation’ (Rose, 1999).

‘..cardiologists in particular were worried that… if they got sent an email with lots of toxic rhythm on it and they didn't respond to say, ‘rush to A&E’ that they would be liable for that patient's wellbeing. That is kind of nonsense, really. Because the patient sees the result first. They have the choice what to do with it’ (Health app designer).

From this perspective, patient organisations acknowledge that health apps play into a broader political agenda, as they could provide the means to monitor one's health and make better choices, replacing the need for direct clinical consultation.

‘What drove a lot of the apps …was also to fill the gaps of what patients were not getting from their healthcare systems... When you get ten minutes talking to a GP or a consultant once a year maybe, what do you do with the rest of the time? You need to monitor and be responsible for your health 365 days a year.’ (Patient organisation representative).

In fact, app designers claim that technologies could help patients play a more active role in medical decision-making. This is because doctors often rely on what patients say about their health, and technology can provide patients with new insights about it.

‘…patients track their health for about one week to ten days, before they go to meet their consultant. They want to go and tell “actually doctor this is how my blood sugar is doing. How do you manage this?” Or in Parkinson's “okay my tremors are more at the end of the day how will you help me”.’ (Health app designer).

Nevertheless, patient organisations are critical of the idea that patients should be collecting data on the false assumption that it would necessarily inform medical decision-making, or that doctors would necessarily use such information.

‘…you can gather all this information and you can send it to your doctor or you can show it to your doctor, …when are they going to have time to read all this stuff? They [doctors] want specific encapsulated information … My blood pressure's going up or going down or whatever. They just need some significant points, don't they? (Patient)

In the context of such views it is important to recognise that patient choice can also amount to the rejection of technology. Patients are not limited to technology in the promotion of their health, nor are they entirely reliant on the options that are prescribed to it. The choice not to self-care technologically is still an option, indicating how neo-liberal healthcare never leaves the individual choice-less (Dean, 1999; McNay, 2009; Vallas and Hill, 2012).

‘…because it may not be my choice to integrate a fit bit that doesn't mean I don't take exercise. It maybe just be that's not my cup of tea.’ (Patient organisation representative).

This section has shown how digital technology renders patients responsible for actively engaging with the production, interpretation and enactment of health data and empowered to get involved in clinical decision-making, suggesting a decentring of medical knowledge. The agential effects of technology however become more evident when patients realise the potential of technology to communalise health knowledge.

### 5.3 The agential potential of technological self-care: ‘health-making’ agency

Our final section discusses the agential potential that emerges within the space between the discourses of empowerment and self-discipline. This form of agency is crystallized in the ways patients become involved in the production of new forms of health knowledge, through their use of digital technologies, and in the ways knowledge is used a) to meet care needs, b) to exchange online peer advice, and c) to inform improvements in healthcare delivery. These three manifestations of patients' agency further illustrate a decentring of health knowledge and, significantly, communalisation of health that counters the more individualizing potential of health technologies. We suggest this constitutes a ‘health-making’ agency.

Our study finds that some patients and carers are directly involved in the development of digital health interfaces, which for some had an entrepreneurial quality. An app developer described their patient collaborators as ‘patient entrepreneurs’ (Crawshaw, 2012; Lupton, 2016b) because they combined strong entrepreneurial engagement with a very high level of contextual and communal expertise concerning their health condition.

‘People are building apps from any age... They are doing it for different reasons. Some are doing it for loved ones. Some are doing it for themselves. …My Sugar is developed by … who doesn't really have too much software expertise. But he has Type 1 Diabetes and he built it with friends and other people that have Type 1 Diabetes. I think the reason why they are getting it right is because they need to use it every day’ (Patient organisation representative).

The ability to uniquely capture, aggregate and share elements of a patient's lived health experience through technological devices enables the production of communal health knowledge that informs both the management of disease and the re-design of services. In other words, this patient-led, technology mediated, expertise creates opportunities for self-care and enables the communal promotion of health. This represents a novel body of knowledge that emanates outside of established biomedical and clinical boundaries, and has the potential to exceed what an individual doctor could possibly know.

‘It's getting away from that paternalistic thing which is the doctor knows best… how can a doctor know about every disease when actually someone can sit at home and probably find out more about a disease than any doctor?’ (Health app designer & doctor).

‘… health is the only thing that all of us have. ... we all live it 24 hours a day. … We are all experts’ (Patient organisation representative).

Extending this point, our study indicates that patients often make decisions about their health on the basis of information shared online by other patients. This resonates with literature across several disciplines - including STS, marketing, information systems - on knowledge exchange in online patient communities (See: Barrett et al., 2016; Foster, 2016; Gilbert, 2016; Johnston et al., 2013; Keeling et al., 2013). Trust in peers has been a significant effect of digital health technology leading to forms of ‘crowd-diagnosis’ and has given rise to the popularity of patient platforms such as *Patients Like Me*, *Patients Know Bes*t, *Care Opinion*, and *Health Unlocked*. While these platforms often pre-determine the type of information people can add (Tempini, 2015), they nonetheless provide a medium for patients to access and contribute to healthcare knowledge outside of the official healthcare sector. This reveals how patients can gradually challenge medical expertise through the communalization of knowledge via digital technology.

‘People trust peer recommendations a lot more than they trust those from healthcare professionals and even pharmaceutical companies people trust even less. But when you get patients saying, this is what I've done and this is how I am managing my diabetes, you say, okay, if they are doing it maybe I can do it.’ (Patient organisation representative)

This critiques the idea that health is an individual matter, and leads towards an appreciation of the collective nature of health. Our study highlights the potential of technological self-care to encourage communalization of health, rendering health a product of collective digital labour.

‘Healthcare is a partnership... I manage my health in partnership with my girlfriend... my mum, my doctor and my dietician, the pharmaceutical company that produces the drugs I take every day...We talk a lot about self care, self management. It's bollocks. … self-care is about knowing where in the system you can support yourself and who in the system can support you as a partnership’ (Patient organisation representative).

Our findings also show that online patient posts could have quasi-therapeutic effects for both the author and readers. Knowing that your story has been read and that someone may sympathise can have positive effects for alleviating traumatic experiences.

‘a health service user posted her story about a crisis service and what she found was that hundreds of people were reading her story. She could see that from the statistics we provide. And that made her feel that her story was important that it mattered to other people and that made her feel better about herself and she tells us that that stopped her from self harming, because she felt that other people were interested in the difficulties she was having with her crisis service. And actually she became somebody who wanted to try and change the service and improve it for other people’ (Patient platform representative)

Patients' experiences are also used for pedagogical purposes, specifically to train nursing, midwifery and paramedic students, improving healthcare and the health of the community further. In this way, future healthcare professionals are exposed to patient concerns and become better equipped to handle them in the future.

‘Staff use the stories that people put there in all kinds of ways. We are seeing them used in teaching as well; about 3000 students are using the site to look at patient experiences’ (Patient platform representative).

## 6 Discussion

This paper examines how policy makers, digital health experts and patient organisations respond to recent policy attempt to encourage patient self-care by means of digital technology (apps and online platforms). Our findings show that digital health technology combines elements of patient empowerment and simultaneous (self-)discipline. We suggest health policy discourse on patient empowerment and self-care has, at its core, an economic rationality and is inextricably linked to a discourse of patient responsibilisation. Responsibility for self-care becomes equated with responsibility for (sustaining) the economic viability of health services and thus becomes implicated in parallel societal discourses around good citizenship (Rose, 2007).

Our findings also show that this health enterprising discourse gives rise to other clinical and societal discourses produced by the possibilities for technology to create additional social value (for instance by improving treatment and learning). Central to the creation of social value is patients' involvement in the production of self-reported health-data that leads to new knowledge for health categorisation and surveillance; this suggests a de-centering (albeit not elimination) of medical expertise. Patients' involvement in the production of new health knowledge by means of digital technology corrects and enriches, and in all cases, challenges medical expertise, reflecting conclusions reached by other studies (Barrett et al., 2016; Griffiths et al., 2012; Novas and Rose, 2000; Radin, 2006; Tempini, 2015). Despite its empowering effects, patients develop this expertise in response to an enterprising health discourse that renders them in charge of interpreting health data and adopting healthy behaviours in line with norms inscribed into the technology algorithmically.

Significantly, the tension between empowerment and discipline creates space for health-making agency. This agency corresponds to the expectations of an enterprising patient identity (in the sense that individuals are expected to use digital technology to self-care) and is thus immanent to a health enterprising discourse. However, it goes beyond this enterprising subjectivity to produce outcomes that can benefit the broader health community, such as the production of new patient-led apps, online peer advice and crowd-diagnosis, healing effects through online sharing of experiences, contribution to clinical research and learning opportunities to health providers.

Foucauldian healthcare scholarship (Ferlie et al., 2012; Hasselbladh and Bejerot, 2007; Waring, 2007; Waring et al., 2016) has typically emphasized the (self-) disciplinary effects of a neoliberal enterprising subject, and with a few exceptions (Martin and Waring, 2018; McGivern et al., 2017) downplayed the potential for agency. Our study suggests that a governmentality reading of technological self-care needs to look beyond its disciplinary effects (Crawshaw, 2012; Martin et al., 2013; McNay, 2009; Randall and Munro, 2010; Skinner, 2013; Waring, 2007) towards new forms of human agency in the use of technology. We suggest the (self-)disciplinary effects of ‘technological self-care’ does not capture the whole extent of its implications. Rather, this dynamic relationship between empowerment and (self-)discipline - inherent in the concept of governmentality (Foucault, 2008, p.64) and evidenced in the use of digital health technology (see Lupton, 2014; Ruckenstein, 2014; Till, 2014) - creates tensions in contemporary healthcare which are manifest in the opportunities for ‘health-making’ agency.

The discourse of technological self-care creates a space for the production of an enterprising patient identity that does not only discipline itself to the expectations embedded in this identity but also becomes involved in the production and dissemination of new health knowledge as part of a broader community. This form of a health-making agency encourages a decentering of health knowledge (from medical authorities to patients) and its subsequent communalization (dissemination of patient knowledge to the broader community) with wider ramifications for the community. We argue that this form of agency is not prescribed into the enterprising patient identity, but operates in its margins or in the interstitial spaces between self-care discourses. Given the ubiquitous nature of technology in the developed world we anticipate similar agential forms of action to emerge in other healthcare contexts.

The study did not trace outright resistance to the introduction of digital health technology for self-care. Given the range of participants in our study (health policy makers, patient organisations, digital technology experts) patients' and doctors' use of digital technology was projected rather than represented. Nevertheless, the agential potential could be substantially circumscribed by doctors' resistance to engage with health apps as suggested by recent research (see: Iacobucci, 2017). Studies on doctors' and patients' use of digital health technology for self-care are thus needed to assess the conditions under which a health-making agency can be realised. We also recognise that different technologies afford different opportunities for agency. For example, health apps intended for clinicians' use and prescribed to patients for monitoring of specific indicators would provide limited opportunities for agency compared to apps or platforms where patients could freely use in many different ways. Further research is required to unpack the forms of agency specific digital health technologies afford according to their purpose, frequency of use and types of data collected.
